# Survey data on dysfunctional attitudes, personality traits, and agreement with persuasive techniques

**DOI:** 10.1016/j.dib.2023.109473

**Published:** 2023-08-05

**Authors:** Annye Braca, Pierpaolo Dondio

**Affiliations:** Technological University Dublin, Ireland

**Keywords:** Dysfunctional attitudes, Personality traits, Persuasion, Marketing, Communication, Cognitive bias

## Abstract

Persuasion techniques play a vital role in human communication, influencing various aspects of our lives. With the increasing prevalence of digital platforms, these techniques have permeated online spaces such as websites, mobile apps, games, and social media. This article presents a dataset collected via a survey, designed to gather information about individuals' demographics, personality traits, dysfunctional attitudes, and their responses to statements embedded with persuasion techniques. Core messages promoting paid news subscriptions, blood donations, and exercise serve as the focus, while definitions and examples of persuasive techniques are provided. By analyzing this comprehensive dataset, researchers could gain valuable insights into the influence and impact of persuasive communication strategies.

**Specifications Table**

**Table:** 

Subject	Social and Personality Psychology
Specific subject area	Persuasion research in social psychology
Type of data	Data relating to a participant's demographic details, personality traits, dysfunctional attitudes and responses to statements that contain embedded persuasion techniques. The dataset is in an excel file.
How the data were acquired	The survey was deployed using Qualtrics [1]*and participants were recruited via the crowd-sourcing marketplace Prolific Academic*[2]. Participant information was collected relating to demographic, personality (Ten Item Personality Inventory (TIPI)) [3], attitudinal (Dysfunctional Attitude Scale (DAS)) [4] and their responses to persuasive statements based on Braca and Dondio [5].
Data format	Raw
Description of data collection	To ensure high quality data from the survey, three attention questions were included to confirm that participants were engaged. Participants were provided with instructions requesting them to answer all questions as honestly as possible. They were also given the instruction: “Please answer each of the questions as you feel right now". Inclusion criteria of the survey required the participants to be over 18 years old, native English speakers, or non-native with high proficiency. There were 2022 survey participants in total with 1995 participants fully completing the experiment. Twenty-five submissions were excluded as these participants failed two of the three attention questions, completed the survey in an unreasonably short time or did not fully complete the survey. As indicated, the final survey dataset consisted of 1995 participant submissions. The questionnaire is provided as a supplementary file.
Data source location	Country: UK, Ireland, USA, Canada, New Zealand and Australia.
Data accessibility	Repository name: Braca, Annye; Dondio, Pierpaolo (2023), “Survey Data on Attitudes: Dysfunctional Attitude Scale (DAS), Ten-Item Personality Inventory-(TIPI), Demographic Information, and Persuasive Techniques”, Mendeley Data, V2, doi: 10.17632/6jpdxs8p2y.2 https://data.mendeley.com/datasets/6jpdxs8p2y

## 1 Value of the Data

• Researchers and policymakers, specifically social scientists in the area of business, economics, and psychology may benefit from this dataset and find it useful for a variety of analyses such as susceptibility to online influence, studies on depression, and dysfunctional thinking.
• The utilization of the dataset has the potential to aid researchers in the development of machine learning and classification models aimed at identifying indicators of depression, maladaptive attitudes, and personality traits. Furthermore, the dataset can facilitate statistical and machine learning analyses concerning the factors that contribute to an individual's vulnerability to online scams.
• Data may provide new insights between dysfunctional attitudes, personality traits, and susceptibility to persuasion techniques.
• Data may serve as region-of-interest for studies of depression and may help in finding possible associations between dysfunctional attitudes and personality traits.
• Data may also be useful to analyze gender/age/location differences in dysfunctional attitudes and personality traits.

## 2 Objective

A large body of research has investigated the link between an individual's psychometric profile and persuasion techniques [6]. The systematic study of persuasion requires assessing an individual's attitudes. Attitudes can be defined as factors such as personality traits, values, and beliefs [5]. The fundamental premise is that different people react differently to persuasion techniques and, as such, it may be possible to use information about a person to predict how they will respond to messages that contain persuasive elements in addition to a core message.

Persuasive language is used for different motives whether to help to sell products or services, or to convince people to accept a view or idea. For example, politicians often use persuasive techniques to influence their audience to agree with their views on a particular topic [5].

We have gathered data relating to survey participants including demographic, personality [3] and belief system-based (DAS) [4]. In this dataset, the response variable is a score given by participants which indicates the level to which they felt convinced by a presented statement. The statement contained a core message such as the benefit of exercise, subscription news and blood donations. All statements were designed following Braca and Dondio [6] guideline examples.

## 3 Data Description

### 3.1 Demographics

The first section of the survey had three questions which identify the individual's age, gender and education level. Table 1 presents a concise overview of the summary and frequency distribution of these demographic variables.

**Table 1:** Description and frequency distribution of demographic variables.

	Frequency	Percent
**Age**		
26–35	749	37.5
36–45	249	12.5
46–55	97	4.9
56–65	37	1.9
Over 60	22	1.1
Under 26	841	42.2
**Gender**		
Man	941	47.17
Non-Binary or Gender Diverse	108	5.41
Woman	946	47.42
**Education**		
Associate Degree (e.g., AA, AS)	100	5
Bachelor's Degree (e.g., BA, BS)	778	39
Doctorate or Professional Degree (e.g., MD,	38	1.9
High School Degree or Equivalent (e.g., GED)	390	19.5
Less Than a High School Diploma	29	1.5
Master's Degree (e.g., MA, MS, MEd)	223	11.2
Some College, No Degree	437	21.9

### 3.2 About personality traits

In this study, participants were administered the Ten Item Personality Measure (TIPI) to assess their Big Five personality traits [7]. The TIPI consists of 10 items that describe different personality traits. Participants were instructed to rate themselves on each trait using a 7-point Likert scale ranging from “Disagree strongly” to “Agree strongly.” These ratings were then converted into numerical scores between 1 and 7 [8]. To facilitate this conversion, the Likert scale was replaced with numerical values as follows: Disagree strongly=1, Disagree moderately=2, Disagree a little=3, Neither agree nor disagree=4, Agree a little=5, Agree moderately=6, Agree strongly=7.

Reverse question keys were employed to score the TIPI scale. Specifically, the following items were reverse scored: Extraversion: 1, 6R; Agreeableness: 2R, 7; Conscientiousness; 3, 8R; Emotional Stability: 4R, 9; Openness to Experiences: 5, 10R [8].

### 3.3 About dysfunctional attitude scale (DAS)

Dysfunctional Attitude Scale (DAS) by Arlene Weissman has been designed to capture individuals' attitudes or beliefs [4]. The instrument consists of 35 questions that aim to measure beliefs associated with Approval, Love, Achievement, Perfectionism, Entitlement, Omnipotence, and Autonomy. DAS questions are scored on a 5-point Likert scale, where the answer to each question is mapped to a DAS scoring key: Agree Strongly: −2, Agree Slightly: −1, Neutral: 0, Disagree Slightly: +1, Disagree Very Much: +2.

The scores obtained from each answer are summarized for each attitude cluster. The first five questions correspond to the Approval attitude (1–5), followed by Love (6–10), Achievement (11–15), Perfectionism (16–20), Entitlement (21–25), Omnipotence (26–30), and Autonomy (31–35). Fig. 2 illustrates the attitude clusters, where values range from −10 to 10, reflecting the sum of the five corresponding questions per attitude. A negative score indicates an area where individuals may be emotionally vulnerable, while a positive score represents an area of psychological strength.

According to Burns [4], these clusters represent an individual's personal philosophy. Therefore, the DAS serves as a valuable tool in capturing individuals' attitudes and beliefs.

### 3.4 Statements with embedded persuasive technique

In this study, we investigated persuasive techniques in three different contexts. Context 1 focused on promoting subscriptions to online news that comply with professional standards and ethics. Context 2 aimed to encourage blood donations. Lastly, Context 3 aimed to motivate individuals to engage in daily walks. Each context presented unique challenges and opportunities for applying persuasive techniques effectively.

We present Table 2 with definitions of these techniques, along with examples from the third context (promoting daily walks). The examples demonstrate how different language styles and embedded cues can effectively convey messages employing specific persuasive techniques.

**Table 2:** Persuasive techniques examples and definitions.

Technique	Definition	Example
Social proof	*This is a phenomenon where decision-making becomes credible and validated through the behaviour of others. People often look to others for clues concerning the correct behaviour - "Wisdom of the Crowds". Social proof is prominent on social networks such as Twitter, Facebook, Instagram and YouTube. The number of followers, fans, views, likes, favorites and even comments that users make can affect how others perceive things*[5].	*“Most people recognize the numerous benefits of being active - consider getting up and going for a healthy walk”*
Authority	*People often tend to comply with requests made by authority figures such as government leaders, law-enforcement representatives, doctors, lawyers, professors, and other perceived experts in different fields. The implicit assumption is that those in positions of authority may wield greater wisdom and power, and therefore, complying with them will lead to a favourable result*[5]*.*	*“Medical experts suggest that people should, at the very least, go for a daily walk which could help to prevent disease and even prolong life - try taking a healthy walk today”.*
Flattery	*The flattery technique uses compliments to entice individuals. The technique boosts a person's sense of identity, helping them feel good about themselves due to the admiration of the flatterer*[5]*.*	*“Smart, successful people like you recognize that small wins matter. Standing up and walking around during the workday can lift your mood and improve your focus and attention.”*
Pathos + Awareness words	*The goal is to evoke an emotional response and to help to gain acceptance, increase responsiveness, and embed ideas and suggestions. Typical persuasive cues to use in this technique are words such as notice, see, realize, aware, experience, discover, consider, contemplate, think about, what if and imagine*[5]*.*	*“People are now less active, sitting more, are screen-fatigued, and are more socially isolated. We need (the worldneeds) to get back to basics. Consider taking a minute and go for a walk.*
Rhetorical question	*The use of this device stimulates critical thinking and encourages drawing out ideas and underlying presuppositions. The goal is to prompt further thinking and reflection*[5]*.*	*“Fitter, healthier, happier - who wouldn't want that? - get up for a minute and go for a walk”*
Antanagoge	*Antanagoge is a rhetorical device that works where a negative point is balanced with a positive point, for example, the popular phrase “When life gives you lemons, make lemonade”. To use this technique, the writer uses deflection - taking the negative aspect of something and pointing out its positive qualities*[5]*.*	*“People are busier than ever and often don't have time for exercise. Yet, you can always go for a walk which is good for your body and mind - try taking a nice walk today.”*
Logic	*Appeal to the audience's sense of reason or logic. To apply this technique, the writer makes clear, logical connections between ideas and utilizes facts and statistics. Using historical and literal analogies to make a logical argument is another strategy*[5]*.*	*“Walking is a simple, fun and free way to get active. It is common sense to engage in healthy exercise. Take a few minutes and go for a walk”.*
Repetition Priming	*Repetition or semantic priming technique helps the audience to see a pattern and be familiar with ideas or words. People typically like things that are familiar to them, with the act of repetition restating and reassuring an idea. As a result, the audience will pay more attention and remember*[5]*.*	*“Get active! Get sweaty! Get up for a minute and go for a walk”*
Anaphora	*The rhetorical device anaphora employs the repetition of words at the beginning of a phrase. The same word/phrase is repeated initially in two successive sentences*[5]*.*	*“Walking is simple, walking is free, and it is one of the easiest ways to become healthier - take a few minutes and go for a walk”*
Framing	*Positive framing is a technique that aims to shape audience perception by emphasizing the benefits, solutions, and opportunities linked to a subject. It utilizes optimistic language, appealing statistics, and success stories to convey a positive message and create a favourable view*[5]*.*	*“Walking can offer numerous health benefits to people of all ages and fitness levels. It may also help prevent certain diseases andeven prolong your life.”*

According to Teodorescu [9] persuasive message design may require breaking the rules of English language grammar and conventions of language usage, and making use of incorrect spelling, neologisms, puns, rhymes and semantic deviations, amongst others. We employed the guidelines established by Braca and Dondio [5] as a foundation for crafting our examples within each domain. By aligning our examples with these established guidelines, we aimed to ensure consistency and relevance in demonstrating the application of persuasive message design.

***Descriptive statistics score persuasive statements***.

## 4 Experimental Design, Materials and Methods

The survey was deployed using Qualtrics software (qualtrics.com) in August 2022. It contains four sections i.e., demographic information, TIPI personality traits (Gosling et al., 2003), DAS attitude information (Burns, 1981) and the persuasion tasks (presented statements for rating). Participants were recruited via the crowd-sourcing marketplace Prolific (prolific.ac.uk). Inclusion criteria required participants to be native English speakers or non-native with high proficiency. Participation was limited to the UK, Ireland, Australia, Canada, USA and New Zealand. In addition, attention questions were included in the survey to ensure that participants were engaged.

### 4.1 Assessing persuasiveness of statements with embedded persuasive technique

**Fig. 1:**
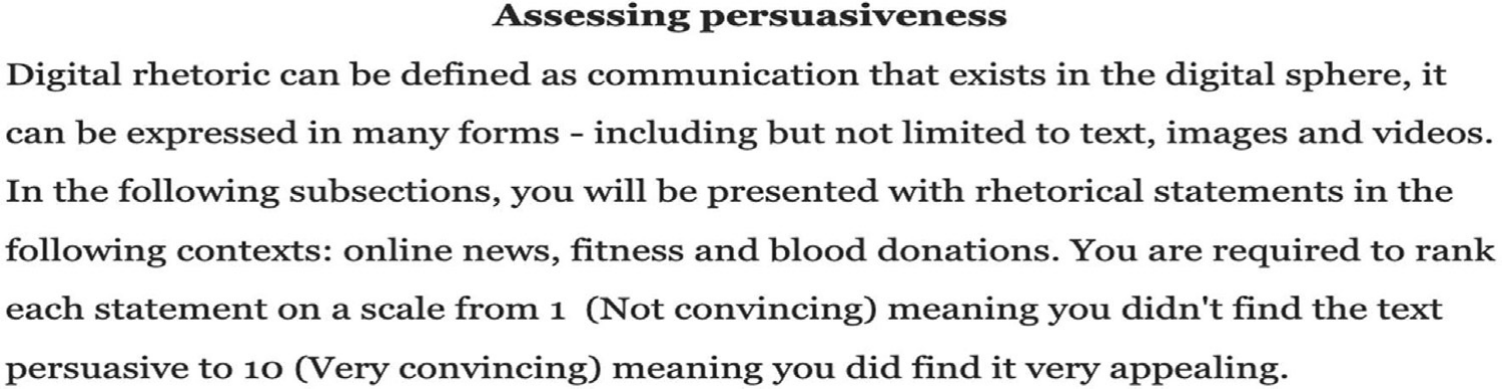
Screenshot of survey instruction to participants.

To measure the influence on participants due to the embedded persuasion techniques present in statements, a Net Promoter Score [12] was adopted where the participant indicates their level of agreement with each statement - survey participants score each statement on a scale from 1 (no effect) to 10 (very convincing).” (See Fig. 1).

**Fig. 2:**
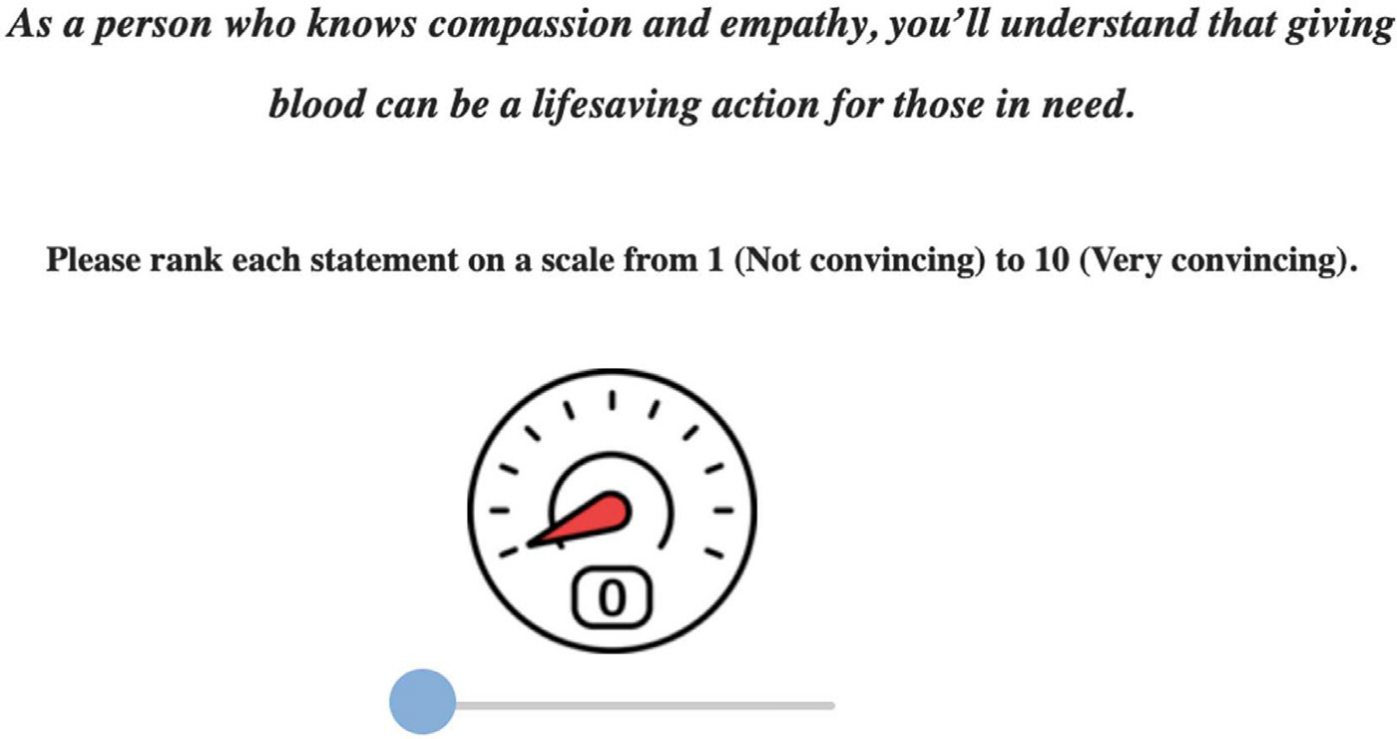
Screenshot of an example of a persuasive statement in survey.

Statements containing a core message aimed to persuade participants about the benefits of subscriptions to paid news services, blood donations, and daily exercise. - with multiple variants being presented that each had an embedded persuasion technique accompanying the core message content. These self-reported scores (called influence scores) formed the response variable. Table 3 showcases descriptive statistics regarding the scores ascribed to the persuasive statements.

**Table 3:** Means and standard deviations of persuasion techniques in three contexts.

Persuasion Technique	Means and Standard Deviations of Persuasion Techniques in Three Contexts					
	Subscribe to Online News		Blood Donations		Daily Exercise	
	Mean	Std. Deviation	Mean	Std. Deviation	Mean	Std. Deviation
Authority	5.46	2.63	7.34	2.23	7.37	2.15
Social Proof	3.84	2.38	7.16	2.39	6.43	2.58
Flattery	5.08	2.57	7.19	2.77	6.77	2.75
Logic	5.23	2.67	7.07	2.49	6.75	2.54
Framing	5.58	2.54	8.66	1.86	7.73	2.27
Pathos	5.40	2.51	7.59	2.2	6.8	2.5
Priming Repetition	4.81	2.78	7.16	2.59	6.11	2.95
Rht. Device Rhetorical Question	4.81	2.72	6.5	2.64	6.56	2.72
Rht. Device Antanagoge	4.61	2.61	6.72	2.65	6.74	2.46
Rht. Device Anaphora	5.23	2.64	7.55	2.32	7.29	2.3

## Ethics Statements

The present study was granted ethical approval by the Technological University Dublin Research Ethics Committee under reference number REC-18-116 for all research activities, including experiments and data collection. Informed consent was obtained from participants through a questionnaire that was presented on the front page. By clicking the ``consent'' button, participants were presumed to have read the survey information and to have voluntarily agreed to participate in the study. As an anonymous online survey was conducted, the authors affirm that all processes that contribute to this research comply with the ethical principles of relevant national and institutional committees for human experimentation, the Helsinki Declaration of 1975, revised in 2000, and the data redistribution policies of the platforms used.

## CRediT Author Statement

**Annye Braca:** Conceptualization, Survey Design, Data Collection, Writing First Draft, Writing Final Draft. **Pierpaolo Dondio**: Conceptualization, survey design, editing final draft.

## Data Availability

Survey data on dysfunctional attitudes, personality traits, and agreement with persuasive techniques. (Original data) (Mendeley Data).

## Appendix Supplementary materials

Image, application 1
