# Supplementary material for: Survey data on dysfunctional attitudes, personality traits, and agreement with persuasive techniques
Source: Data Brief. 2023 Aug 5;50:109473. doi: 10.1016/j.dib.2023.109473 (PMC10440443; doi:10.1016/j.dib.2023.109473)
Supplement: Supplementary file 1 [file mmc1.docx]

Annotate version Survey.

| ***Section I: Questions About Demographics*** |
| --- |
| 1. *What is your age?*  - *26 - 35 (0)* - *36 – 40 (1)* - *46 – 55 (2)* - *56- 65 (3)* - *Under 26 (4)* - *Over 65 (5)*  1. *What gender do you identify as?  [gender]*  - *Female (0)* - *Non-Binary or Gender Diverse (1)* - *Male (2)*  1. *What is the highest degree or level of education you have completed?*  - *Associate Degree (e.g., AA, AS) (0)* - *Bachelor's Degree (e.g., BA, BS) (1)* - *Doctorate or Professional Degree (e.g., MD) (2)* - *High School Degree or Equivalent (e.g., GED) (3)* - *Less Than a High School Diploma (4)* |

| ***Section II: Questions about personality traits (TIPI)*** | |
| --- | --- |
| *Preliminary Information*  *For each of the 10 questions below, declare how you see yourself using the following scale:*  *1 = Disagree strongly*  *2 = Disagree moderately*  *3 = Disagree a little*  *4 = Neither agree nor disagree*  *5 = Agree a little*  *6 = Agree moderately*  *7 = Agree strongly* | *I see myself as:*  *1. Extroverted, enthusiastic. (TIPI1)*  *2. Critical, quarrelsome. (TIPI2)*  *3. Dependable, self-disciplined. (TIPI3)*  *4. Anxious, easily upset. (TIPI4)*  *5. Open to new experiences, complex. (TIPI5)*  *6. Reserved, quiet. (TIPI6)*  *7. Sympathetic, warm. (TIPI7)*  *8. Disorganised, careless. (TIPI8)*  *9. Calm, emotionally stable. (TIPI9)*  *10. Conventional, Uncreative (TIPI10)* |
| *TIPI scale scoring (“R” denotes reverse-scored items):*  *Extraversion: 1, 6R; Agreeableness: 2R, 7; Conscientiousness; 3, 8R; Emotional Stability: 4R, 9; Openness to Experiences: 5, 10R.*  ***Section III: DAS Scale Questionnaire*** | |
| *Answering this section is quite simple, put a check in the column that represents your estimate of how you think most of the time. Be sure to choose one answer for each attitude. Because we all are different, there is no “right” or “wrong” answer to any statement. To decide whether a given attitude is typical of your own philosophy, recall how you look at things most of the time.* | |
| *1. Agree Strongly (-2)*  *2. Agree Slightly (-1)*  *3. Neutral (0)*  *4. Disagree Slightly (1)*  *5. Disagree Very Much (2)* | |
| *Answer the following questions:* | |
| *1. Criticism will obviously upset the person who receives the criticism. (DAS1)* | |
| *2. It is best to give up my own interests in order to please other people. (DAS2)* | |
| *3. I need other people’s approval in order to be happy. (DAS3)* | |
| *4. If someone important to me expects me to do something, then I should do it. (DAS4)* | |
| *5. My value as a person depends greatly on what others think of me. (DAS5)* | |
| *6. I cannot find happiness without being loved by another person. (DAS7)* | |
| *7. If others dislike you, you are bound to be less happy. (DAS8)* | |
| *8. If people whom I care about rejects me, it means there is something wrong with me. (DAS9)* | |
| *9. If a person I love does not love me, it means I am unlovable. (DAS10)* | |
| *10. Being isolated from others is bound to lead to unhappiness. (DAS11)* | |
| *11. If I am going to be a worthwhile person, I must be truly outstanding in at least one major respect. (DAS12)* | |
| *12. I must be a useful, productive, creative person or life has no purpose. (DAS13)* | |
| *13. People who have good ideas are more worthy than those who do not. (DAS14)* | |
| *14. If I do not do as well as other people, it means I am inferior. (DAS15)* | |
| *15. If I fail at my work, then I am a failure as a person. (DAS16)* | |
| *16. If you cannot do something well, there is little point in doing it at all. (DAS17)* | |
| *17. It is shameful for a person to display their weaknesses. (DAS18)* | |
| *18. A person should try to be the best at everything they undertake. (DAS19)* | |
| *19. I should be upset if I make a mistake. (DAS20)* | |
| *20. If I don’t set the highest standards for myself, I am likely to end up a second-rate person. (DAS21)* | |
| *22. It is necessary to become frustrated if you find obstacles to getting what you want. (DA22)* | |
| *23. If I put other people’s needs before my own, they should help me when I need something from them. (DAS23)* | |
| *24. If I am a good husband/wife (or partner), then my spouse/partner is bound to love me. (DAS24)* | |
| *25. If I do nice things for someone, I can anticipate that they will respect me and treat me just as well as I treat them. (DAS25)* | |
| *26. I should assume responsibility for how people feel and behave if they are close to me. (DAS26)* | |
| *27. If I criticize the way someone does something and they become angry or depressed, this means I have upset them. (DAS27)* | |
| *28. To be a good, worthwhile, moral person, I must try to help everyone who needs it. (DAS28)* | |
| *29. If a child is having behavioural difficulties, this shows that the child’s parents have failed in some important respect. (DAS29)* | |
| *30. I should be able to please everybody. (DAS30)* | |
| *31. I cannot expect to control how I feel when something bad happens. (DAS31)* | |
| *32. There is no point in trying to change upsetting emotions because they are a valid and inevitable part of daily living. (DAS32)* | |
| *33. My moods are primarily created by factors that are largely beyond my control, such as the past, body chemistry, hormone cycles, biorhythms, chance or fate. (DAS33)* | |
| *34. My happiness is largely dependent on what happens to me. (DAS34)* | |
| *35. People who have marks of success (good looks, social status, wealth, fame) are bound to be happier than those who do not. (DAS35)* | |

| ***Section IV Assessing Persuasiveness*** |
| --- |

- *Domain 01: Encouraging the adoption of online news subscriptions that adhere to professional standards and ethical principles.*
- *Domain 02: Promotion of blood donation.*
- *Domain 03: To motivate individuals to take a daily walk outside.*

*t= technique*

*d= domain*

- 1. *“News and journalism generate important benefits for society through the production and dissemination of knowledge. Subscription-driven news could promote a shift in focus from quantity to quality of content that complies with professional standards and ethics.” [Technique – Positive Framing (Main-argument-context-positive-framing-domain1: (t1_d1))]*
  2. *“Donating blood saves lives. Blood donations keep people alive in emergency situations and can improve the quality of life through the treatment of chronic conditions. Giving blood makes it possible for many people to lead normal healthy lives.” [Technique - Positive Framing (Main-argument-context-positive-framing-domain2- (t1_d2))]*
  3. *“Walking can offer numerous health benefits to people of all ages and fitness levels. It may also help prevent certain diseases and even prolong your life.” [Technique -Positive Framing (Main-argument-context-positive-framing-domain3 (t1_d3))]*
  4. *“In a recent survey, the majority of people said that they are willing to pay for news subscriptions – most people are subscribing to news services.” [Technique - Social-proof-1 (t2_d1)]*
  5. *“Each day, thousands of people around the world donate blood providing a vital service to the community - consider giving blood to help others.” [Technique - Social-proof-2(t2_d2)]*
  6. *“Most people recognize the numerous benefits of being active - consider getting up and going for a healthy walk.” [Technique - Social-proof-3(t2_d3)]*
  7. *“As an open-minded person who knows the value of critical thinking, you will likely appreciate the quality journalism that most subscription-driven news sites provide.” [Technique - Flattery-1 (t3_d1)]*
  8. *“As a person who knows compassion and empathy, you’ll understand that giving blood can be a lifesaving action for those in need.” [Technique - Flattery-2 (t3_d2)]*
  9. *“Smart, successful people like you recognize that small wins matter. Standing up and walking around during the workday can lift your mood and improve your focus and attention.”[ Technique - Flattery-3 (t3_d3)]*
  10. *“High-quality journalism should be supported, don’t you think? - consider subscribing to a quality news service.” [Technique - Rht-Question-1 (t4_d1)]*
  11. *“Did you know that helping others can lead to a happier, healthier you? Consider giving blood to help those in need.” [Technique - Rht-Question-2 (t4_d2)]*
  12. *“Fitter, healthier, happier - who wouldn’t want that? - get up for a minute and go for a walk.” [ Technique - Rht-Question-3 (t4_d3)]*
  13. *“Free news content is often less valuable and trustworthy than news you pay for. Thankfully, publishers who produce truly distinct, valuable and trusted journalism can be found online”. [Technique - antanagoge-1 (t5_d1)]*
  14. *“For some people needles can be scary and the sight of blood can be intimidating. However, blood donations keep people alive in emergencies and are critical for the treatment of chronic conditions.” [Technique - antanagoge-2 (t5_d2)]*
  15. *“People are busier than ever and often don’t have time for exercise. Yet, you can always go for a walk which is good for your body and mind - try taking a nice walk today.” [Technique - antanagoge-3 (t5_d3)]*
  16. *“It is common sense to avoid misinformation and poor-quality content. Notice that by subscribing to trusted sources, people can enrich their knowledge and awareness.” [Technique - logic-1 (t6_d1)]*
  17. *“Donating one unit of blood may save the lives of up to three people. It is wise and common sense to support our communities - blood shortages will affect nearly everyone.” [Technique - logic-2 (t6_d2)]*
  18. *“Walking is a simple, fun and free way to get active. It is common sense to engage in healthy exercise. Take a few minutes and go for a walk.” [Technique - logic-3 (t6_d3)]*
  19. *“UNESCO has expressed concern about the rise of online misinformation. Reading premium content and paying for our news could be a way to ensure the quality of content and the benefits that go with access to trusted journalism.” [Technique - authority-1 (t7_d1)]*
  20. *“According to the American Red Cross, at least 25% of us will need a blood transfusion at least once in our lifetime. Health service authorities appeal to individuals to give blood to help those in need.” [Technique - authority-2(t7_d2)]*
  21. *Medical experts suggest that people should, at the very least, go for a daily walk which could help to prevent disease and even prolong life - try taking a healthy walk today. [Technique - authority-3(t7_d3)]*
  22. *“Good quality journalism generates important benefits for society through the dissemination of knowledge. Think about supporting real journalism and consider getting a news subscription.” [Technique - pathos-1 (t8_d1)]*
  23. *“Donating blood is one of the most personal and generous ways to help others - your gift will impact people in ways you can’t even imagine. Think about giving blood to help those in need.” [Technique - pathos-2 (t8_d2)]*
  24. *“People are now less active, sitting more, are screen-fatigued, and are more socially isolated. We need (the world needs) to get back to basics. Consider taking a minute and go for a walk.” [Technique - pathos-3 (t8_d3)]*
  25. *“Always honest, always accurate and always trustful - read the best and subscribe to a quality news service.” [Technique - repetition-1 - (t9_d1)]*
  26. *“Be brave, be kind, be someone's hero - consider giving blood to help those in need.” [Technique - repetition-2 - (t9_d2)]*
  27. *“Get active! Get sweaty! Get up for a minute and go for a walk.” [Technique - repetition-3 - (t9_d3)]*
  28. *“For those who like to read, for those who like quality and for those who like smart content - consider subscribing to a quality news service.” [ Technique - Rht anaphora-1 (t10_d1)]*
  29. *“We cannot always prevent cancer, we cannot always prevent accidents - but we can, at the very least, make sure that no one dies due to lack of blood donors - consider giving blood to help those in need.” [ Technique - Rht anaphora-2 (t10_d2)]*
  30. *“Walking is simple, walking is free, and it is one of the easiest ways to become healthier - take a few minutes and go for a walk.” [ Technique - Rht anaphora-3 (t10_d3)]*
